# Studies on the Pathophysiology and Genetic Basis of Migraine

**DOI:** 10.2174/13892029113149990007

**Published:** 2013-08

**Authors:** Claudia F Gasparini, Heidi G. Sutherland, Lyn R Griffiths

**Affiliations:** Genomics Research Centre, Griffith Health Institute, Griffith University, Gold Coast Campus, Building G05, GRIFFITH UNIVERSITY QLD 4222, Australia

**Keywords:** Migraine, Migraine with aura, Migraine without aura, Familial hemiplegic migraine, Molecular genetics, Genes

## Abstract

Migraine is a neurological disorder that affects the central nervous system causing painful attacks of headache. A genetic vulnerability and exposure to environmental triggers can influence the migraine phenotype. Migraine interferes in many facets of people’s daily life including employment commitments and their ability to look after their families resulting in a reduced quality of life. Identification of the biological processes that underlie this relatively common affliction has been difficult because migraine does not have any clearly identifiable pathology or structural lesion detectable by current medical technology. Theories to explain the symptoms of migraine have focused on the physiological mechanisms involved in the various phases of headache and include the vascular and neurogenic theories. In relation to migraine pathophysiology the trigeminovascular system and cortical spreading depression have also been implicated with supporting evidence from imaging studies and animal models. The objective of current research is to better understand the pathways and mechanisms involved in causing pain and headache to be able to target interventions. The genetic component of migraine has been teased apart using linkage studies and both candidate gene and genome-wide association studies, in family and case-control cohorts. Genomic regions that increase individual risk to migraine have been identified in neurological, vascular and hormonal pathways. This review discusses knowledge of the pathophysiology and genetic basis of migraine with the latest scientific evidence from genetic studies.

## 1. INTRODUCTION

Migraine is a highly prevalent disorder with undetermined cause which is estimated to affect 12% of the Caucasian population including 18% of adult women and 6% of adult men [1, 2]. The prevalence of women suffering from migraine has been attributed to a role of ovarian hormones [3]. The socioeconomic impact of migraine is wide-ranging and long-lasting imposing direct medical costs to individuals, families and communities and indirect costs due to lost productivity at work. The European Community has estimated migraine to cost more than €27 billion per year making it one of the most costly neurological disorders [4].

Migraine typically commences in puberty but has most impact on people in the 35 to 45 age bracket [3]. Migraineurs experience a severe headache with associated symptoms of nausea, vomiting, photo- and phono-phobia [5]. The pain experienced during the headache phase can localise to one side of the head or pulsate and can be worsened by physical activity. The frequency of headache attacks varies, occurring approximately once a week, or once a month to once a year, and in duration from as little as an hour to as much as three days. The variability in frequency of migraine attacks is related to both the genetic component carried by the individual and environmental triggering factors [6]. 

Migraine is diagnosed based on a patient’s recollection of their symptoms, a positive family history and the exclusion of secondary causes [7]. Symptoms are assessed by symptom-based criteria defined by the International Headache Society (IHS), International Classification of Headache Disorders 3rd Edition (beta version currently available at http://cep.sagepub.com/content/33/9/629.full) [8]. Two main types of migraine have been recognized: migraine with or without aura (MA and MO, respectively). Patients with MA experience an aura that precedes their headache, whilst those with MO do not. The aura is experienced by about a third of patients and consists of neurological symptoms that manifest as visual hallucinations like flashing lights, sparks or lines (followed by dark spots), facial tingling or numbness as well as other sensory, motor or aphasic symptoms that usually last from 5 minutes to an hour [9]. Aura has been linked to an electrophysiological event that occurs during migraine, termed cortical spreading depression (CSD) which is a wave of intense neuronal activity that gradually propagates over the cortical regions of the brain and is then followed by prolonged inhibition of neuronal activity [10]. 

## 2. MIGRAINE PATHOPHYSIOLOGY

Migraine pathophysiology has metamorphosed from a disease originally attributed to supernatural causes to a well characterised neurological disorder [11]. Migraine symptoms arise from a combination of vascular and neurological events occurring in the cranial meninges and as a result this disorder is often described as being of ‘neurovascular’ origin because of the two interacting systems [12]. Some key events implicated are the phenomenom of CSD and activation of the trigeminovascular system with neurogenic inflammation, leading to changes in the meningeal vasculature [13]. The exact sequence for these pathological events and how they interact is not clear but what is known is that genetic mutations affect an individuals’ likelihood of developing migraine and that a number of brain structures, including the trigeminal innervations of the cranial vessels, are involved [14, 15]. The following discussion reviews the current understanding of migraine pathophysiology and studies on the genetic basis of the disorder. 

### 2.1. The Vascular and Neurogenic Theories

The exact cause of migraine headache pain is still not completely understood. Historically, two independent theories, the vascular theory and the neuronal theory, explaining the aetiology of migraine headache were proposed. The vascular theory was introduced by Thomas Willis who combined keen clinical observation and meticulous anatomic dissection to redefine thinking about "megrim" [16]. In his vascular theory he recognised that “all pain is an action violated” and argued the pain from headache is caused by vasodilation of the cerebral and meningeal arteries [16]. 

In the 20^th^ century the work of Graham and Wolff advanced the vascular theory. Graham and Wolff were the first people to study headache in the laboratory from the (1930s-1950s) and fostered the idea that migraine is a vascular event mediated by initial intracranial vasoconstriction that is followed by rebound vasodilation [17]. Consistent with this theory is the throbbing quality of pain and the relief patients experienced with administration of Ergotamine, a potent vasoconstrictor [18]. Also the observed headache inducing effects of nitroglycerin, a vasodilator was taken as added proof of migraine's vascular nature [19]. Finally insights into the mechanism of action of the triptans (serotonin receptor agonists) which revolutionised the treatment of migraine, and more recently calcitonin gene-related peptide (CGRP) agonists, suggest that they may primarily relieve symptoms through cranial vasoconstriction [20].

More recently in a study by Amin *et al.*, 2013 using a high-resolution magnetic resonance angiography imaging technique during spontaneous unilateral migraine attacks has shown that although vasodilation coupled with the release of vasoactive substances is a key physiological occurrence in migraine pathophysiology, dilatation is not the cause of peripheral and central pain pathways [21]. The release of neuropeptides and proinflammatory substances from trigeminal afferents in the meninges accompanies vascular changes and initiates the sensation of pain by sensitizing peripheral and central neurons within the trigeminovascular system [22]. These vascular events cannot be ignored and are important in further understanding the full gamut of migraine processes so as to clarify how a migraine episode is initiated and sustained. 

The alternative neurogenic theory centres on migraine being a disorder of the brain where vascular events are best explained by dysfunction of neuronal networks [22]. Migraine is a consequence of neural events which have a strong genetic linkage however the translation of these neural events into migraine pain remains to be explained. Support for the neurogenic theory comes from the fact that certain neurological symptoms that occur during auras cannot be explained by a purely vascular model of headache [23]. This theory focuses on the cause of migraine pain and is currently linked to activation of the trigeminovascular system [22]. The prime component of this system is the trigeminal nerve and its nerve fibers which innervate meningeal blood vessels and other brainstem structures [14]. This theory also implicates the phenomenon of cortical spreading depression (CSD) as the cause of neurological symptoms of aura [24]. Clearly neither theory can account for the entire cascade of events observed and both theories have been integrated in a neurovascular model. 

### 2.2. The Trigeminovascular System

The trigeminovascular system (TGVS) consists of the trigeminal nerve and nerve fibers which innervate the network of extra- and intra- cranial meningeal blood vessels and the brain stem [25]. This system is thought to play an integral role in regulating vascular tone and in the transmission of pain signals. Activation of this system during the pain phase of migraine is thought to initiate a cascade of chemical activity from trigeminal sensory nerve endings [14, 26]. Component trigeminal sensory nerves of this system store several vasoactive neuropeptides including: substance P (SP), calcitonin gene-related peptide (CGRP), neurokinin A, nitric oxide (NO) and pituitary adenylate cyclase-activating peptide (PACAP) that upon being released lead to inflammation and dilation of blood vessels aggravating the pain [27]. Neuropeptides are important molecules that cause vasodilation and increase blood flow leading to edema in the meningeal vasculature as well as an inflammatory response around vascular structures in the meninges which is believed to be responsible for head pain [28]. Precisely the peripheral terminations of the TGVS are located in correspondence of the extracranial soft tissues, such as muscles, eye, ear, skin, subcutaneous tissue, nasal cavities, arteries, periosteum, and also of intracranial structures, or venous sinuses, vagus and glossopharyngeal nerves [22]. 

### 2.3. Cortical Spreading Depression

Cortical spreading depression is an electrophysiological event that was first described in 1943 by Aristides Leão, a Brazilian neurophysiologist doing his doctoral research on epilepsy at Harvard University [29]. Functional neuroimaging studies in human brain have clearly demonstrated changes in blood flow and brain activity in migraineurs that is indicative of CSD [30-32]. CSD is best described as an intense wave that propagates across the cerebral cortex at a rate of 2-5mm/min lasting 15-30mins and that causes disruption of ionic gradients (Ca^2+^, Na^+^, K^+^) followed by a period of suppressed neural activity [33, 34]. Similar events have been reported in stroke and brain injury [24]. CSD has been shown to cause activation and sensitization of the trigeminovascular system, initiating a series of neural, vascular and inflammatory events that result in pain [35]. A recent study has shown that CSD caused Pannexin1 megachannel opening in neurons resulting in caspase-1 activation and HMGB1 release which initiates parenchymal inflammatory pathways and may provide the stimulus for sustained trigeminal activation and lasting pain [36]. CSD is widely accepted to be the cause of visual auras based on experimental evidence from patients and animal models however at present there is no agreement that it is the cause of pain. Although CSD does occur in migraine with aura patients it does not explain the cause of the majority of headaches which occur in 70% of migraineurs. 

### 2.4. Animal Models of Migraine

Animal models have been used for many diseases to study the pathological mechanisms and to test the efficacy of drugs. In migraine the broad clinical phenotype and the fact that multiple genetic and environmental factors play a role in the disorder has made the development of a reliable animal model difficult. Nevertheless a variety of animal models of migraine have been developed to better study the pathophysiology of migraine including activation of the trigeminovascular system, the pain-producing cranial structures, the large vessels, dura mater and electrical stimulation of the trigeminal ganglion to illustrate pain pathways and model migraine symptoms such as allodynia [37]. Many animal models also try to replicate the phenomenon of CSD however none of them come close to replicating all facets of the migraine syndrome [38]. Animals have also contributed to the study of migraine mainly by simulating the effects of headache using different pharmacological compounds [39, 40]. A well validated human migraine model is based on the administration of glyceryl trinitrate (GTN) to simulate the effects of headache [41]. Also structures like *in vitro* blood vessels have served the testing of drugs like triptans and for probing the location of ‘triptan receptors’ in vascular tissue [34]. Additionally Melo-Carrillo *et al.*, 2013 describe a murine model of chronic meningeal nociception that mimics migraine clinical features and propose their technique as a new animal model to study migraine [42]. This model demonstrates an increase in nociceptive behaviours related to headache: rest, facial grooming and freezing as a result of meningeal infusion of inflammatory soup [43]. These models allow the study of migraine *in vivo* and are good tools with which to screen potential pharmaceutical compounds.

## 3. MOLECULAR GENETIC STUDIES OF MIGRAINE

Insights into the genetic basis of migraine have come from a number of different angles. Linkage studies in family pedigrees in which inheritance of migraine is apparent have been used to identify genomic regions, and in some cases the genes, which are responsible for susceptibility. Knowledge gained from studies of migraine pathophysiology has led to investigation of candidate genes in migraine case-control populations and more recently genome-wide association studies in large case-control cohorts have been used to identify genes potentially involved in migraine with no a priori assumptions.

### 3.1. Studies on Familial Hemiplegic Migraine

Familial hemiplegic migraine (FHM), a rare subtype of MA first described by Clarke (1910) in a UK family of 4 generations, has been the object of much interest by geneticists researching migraine [44]. FHM is inherited in an autosomal dominant fashion and some symptoms overlap with those of migraine with aura. FHM symptoms typically include hemiparesis (weakness of half the body) during the aura phase and the aura is generally more prolonged and consists of temporary visual changes such as blind spots (scotomas), flashing lights, zig-zagging lines, and double vision [45]. Familial diagnosis requires that at least one first- or second-degree relative be affected. In cases of identical symptomatology and the absence of affected first- or second-degree relatives, the disorder is classed as sporadic hemiplegic migraine (SHM) [8]. The stronger genetic component and phenotypic overlap of FHM with MA has made FHM a favourable model to study the mechanisms of headache and aura [46]. 

FHM was the first primary headache disorder connected to genetic mutations [44, 47, 48]. Three causative genes have been identified via linkage in FHM families and include: *CACNA1A*, *ATP1A2* and *SCNA1A* (Table **1**). The proteins produced by these genes form channels that regulate the flow of ions across neuronal and glial cell membranes. The common theme that ties these three genes together is ‘ion transport’. The familial forms of hemiplegic migraine identified are referred to as FHM1, FHM2, and FHM3, respectively. Each type of FHM is caused by mutations in a different gene.

The first FHM region (FHM1; MIM141500) was localized to 19p13 and the defective gene *CACNA1A* (MIM601011) was identified [44]. This gene was identified whilst studying families segregating with cerebral autosomal dominant arteriopathy with subcortical infarcts and leukoencephalopathy (CADASIL), (MIM125310) a disorder that causes stroke and vascular impairments which can also cause migraine [49]. The *CACNA1A* gene encodes the α1 subunit of the voltage-dependent P/Q-type Ca^2+^ channel protein [50]. This channel is expressed in neuronal tissue where it regulates the flow of calcium ions Ca^2+^ into excitable cells [51]. Mutations in this gene contribute to cerebellar ataxia and epilepsy and can cause 2 other neurological disorders with autosomal dominant inheritance, episodic ataxia type 2 (EA2; MIM108500), and spinocerebellar ataxia type 6 (SCA6; MIM183086) [50].

The second type of familial hemiplegic migraine, FHM2 (FHM2; MIM602481), is caused by mutations in the gene *ATP1A2* (ATP1A2; MIM182340) [48]. The *ATP1A2* gene encodes the α2 isoform of the major subunit of the Na^+^/K^+^-ATPase pump. Astrocytes are the main cells expressing this type of channel and when mutated ion pumps have higher resting potentials [52]. The third FHM locus (FHM3; MIM609634) is at 2q24, and the implicated gene *SCNA1A* (SCN1A; MIM182389) encodes the α1 subunit of the neuronal voltage-gated Na^+^ channel [53]. This channel is important for action potential generation in neurons. Mutations in *SCN1A* were first observed to cause the epilepsy syndrome, generalised epilepsy with febrile seizures (GEFS+; MIM604233) and severe myoclonic epilepsy of infancy (SMEI; MIM607208) [54, 55]. 

Variants in the three FHM genes, however do not account for 100% of FHM cases and it has been proposed that there are additional mutations at other locations that could potentially contribute to the FHM phenotype. *SLC1A3 *(encoding the glial glutamate transporter *EAAT1*) and *SLC4A4* (encoding the electrogenic sodium bicarbonate cotransporter NBCe1) have been proposed as potential fourth and fifth genes (FHM4 and FHM5) responsible for pure hemiplegic migraine [52, 56]. Functional studies of mutations at each FHM locus in animal and model cells have shown that various missense mutations, large and small scale deletions exist and greatly affect the conductive properties of the channels upsetting the balance of ions in neurons [57]. The flow of ions is critical for normal physiological functioning and any disruption can make people more susceptible to developing these severe headaches. 

### 3.2. Mouse Models of FHM

Genetically modified mice have been engineered based on mutations in the three specific genes responsible for FHM with the expectation that they may by extrapolation reflect on the pathophysiology of common migraine subtypes and also due to the fact that no other genes of strong effect are available and amenable to study common migraine [34]. Despite the large number of mutations characterized at each FHM locus, only a small fraction of mutations that produce very extreme phenotypes have been studied in genetically altered mice given the expense and time involved in generating modified animals [58]. 

Two common FHM1 mutations in the *CACNA1A* gene have been introduced in knock-in mice: R192Q [59, 60] and S218L [61, 62]. Both mutations change the amino acid sequence of the protein and affect the 3D structure and function of the α1 subunit of the voltage-dependent P/Q-type Ca^2+^ channel. The channel mediates the influx of calcium ions Ca^2+^ and changing the amino acid sequence has important consequences to normal physiological functions which can be observed *in vivo*. The R192Q mutation is located in the fourth transmembrane domain and causes a gain-of-function effect. This mutation contributes to excess release of glutamate in the cortex and has been suggested to increase susceptibility to CSD, the physiological event responsible for the aura in migraine [63]. The phenotype of S218L mutation is more extreme than R192Q and mice exhibit symptoms of cerebellar ataxia, seizure and head trauma and in particular increased sensitivity to CSD [62]. The S218L mutation is located in the second intracellular loop of the protein and mice with this mutation exhibit repetitive CSD events after a single stimulus with an increase in Ca^2+^ influx [62]. 

A knock-in mouse carrying the FHM2 mutation W887R in the human *ATP1A2* gene has also been generated. This mutation is located in the fourth extracellular loop between transmembrane domains M7 and M8 of the subunit that codes for the Na^+^-K^+^ATP pump involved in ion transport in the brain and causes the protein to be non-functional and unable to pump ions. Mice homozygous for this mutation do not survive past birth due to neurological dysfunctions, while in heterozygotes CSD occurs more easily and quickly than in wild type mice [64]. Thus FHM1 and FHM2 mouse models have contributed to better understanding the functional properties of Ca^2+^ channels and suggest that relevant mutations reduce the threshold for CSD induction and propagation, supporting a role of CSD in triggering migraine [64].

### 3.3. Studies on Common Migraine

The causation of some diseases can be linked primarily to genetic factors, but is more commonly the result of an interaction between genetic and environmental triggers. Migraine is phenotypically and genetically heterogeneous and no single variant can explain the entire underlying genetic component across different families and populations. The common form of migraine does not follow a Mendelian mode of inheritance making the identification of susceptibility genes more complex. However, there is robust evidence from epidemiological studies in twins, families and unrelated cases of migraine indicating genetics plays a significant part in migraine expression. The results indicate that first degree relatives of migraineurs are RR=1.88 times more likely to suffer migraine than first degree relatives of non-migraineurs [65]. The increased occurrence of migraine in close relatives of an affected individual is known as familial aggregation. At the population level heritability is estimated at 0.40-0.60 [66] with the residual heritability reflecting the environmental component influencing the disorder. The variable phenotypic presentation of migraine and the results of many genetic studies to date suggest that common migraine is polygenic.

### 3.4. Migraine Linkage Studies

To date several linkage studies have been performed utilizing families of different ethnic origin and have successfully identified migraine susceptibility loci on a range of chromosomes demonstrating that migraine is polygenic. The majority of linkage studies have used the ‘migraine end diagnosis’ definition to diagnose migraine patients as either MA or MO. This has worked well for the most part however many loci have not been replicated in populations of different ethnicity. It has been suggested by some that the reason for this occurrence is the presence of rare high-impact family specific markers. Other reasons to explain lack of replication include inaccurate migraine diagnosis and phenotyping of migraine cases leading to heterogeneity of cohorts. 

Two alternative phenotyping strategies to the end diagnosis have been introduced in an attempt to identify novel regions which are more specifically related to migraine biological processes. One strategy utilizes latent classes (LCA) whilst the other examines trait components (TCA). The LCA and TCA strategies introduced in studies by Nyholt, Anttila and colleagues make better use of the questionnaire-based information [67, 68]. Employment of strategies like LCA and TCA have shown certain chromosomal regions to be linked to specific migraine symptoms like pulsating pain, phono-/photophobia, nausea, and age at onset of migraine. One example is the identification of the 5q21 region in an Australian study and the 17p13 region in a Finnish study associated with pulsating head pain through the application of the LCA method by Nyholt *et al.*, 2005 [69, 70]. Also the LCA method identified linkage at 18p11 [70]. These two methods have added somewhat to standard ICHD 3rd Edition (beta version) classification criteria in identifying linkage regions with specific migraine symptoms. 

Out of all the linkage studies done the most consistent locus resides on chromosome 4. This is an interesting candidate region with two independent linkage studies of 50 Finnish MA families showing linkage to 4q24 [67, 71]. Another study using 103 Icelandic families identified an overlapping locus at 4q21 using MO patients only [72]. In a recent study by Oedegaard, *et al.*, 2010 [73] performed in 31 families with bipolar disorder co-morbid with migraine they replicated the 4q24 region implicating this locus as potentially containing a gene predisposing to MA and MO [73]. 

The power of linkage is evident in the study by Lafreniere *et al.*, 2010 who identified the first causal typical migraine gene in a multigenerational family with dominant, fully penetrant typical MA [74]. The *KCNK18* gene at chromosome 10q25.3 encodes a two-pore domain potassium (K2P) channel TRESK (*KCNK18*; MIM613655). In situ hybridization in the mouse embryo detected expression in the trigeminal ganglion, dorsal root ganglia and autonomic nervous system ganglia implicating TRESK in neuronal excitability [74]. Mutations in TRESK were identified by Sanger sequencing the coding region of 110 unrelated migraineurs and 80 controls. The most interesting variant identified was a frameshift (F139WfsX24) mutation which prematurely truncates the protein and results in a total loss of channel function [75, 76]. Further gene sequencing in an Australian cohort (511 migraine, 505 controls) identified nine additional variants in this gene. The results of these studies indicate functional variants in the *KCNK18* gene may be involved in MA pathogenesis by lowering the threshold for CSD. The fact that the gene identified codes for an ion channel strengthens the hypothesis of neuronal hyperexcitability and provides a novel target for the pharmaceutical industry.

## 4. CANDIDATE GENE STUDIES

The goal of molecular genetics studies is to find a genetic abnormality that may predispose an individual to a specific disease. This problem is approached by identifying the physiological mechanism causing disease and then looking for a disrupted biochemical product at the protein level that can be exploited pharmacologically or diagnostically [77]. Genetic efforts have focussed on candidate genes involved in pathological pathways of the disease and have examined genes involved in neurological, vascular, hormonal and more recently mitochondrial functions. 

### 4.1. Neurological Pathways

Although D’Onofrio *et al.*, 2009 [78] found that a combination of two SNPs in the *CACNA1A* gene may contribute to migraine susceptibility, polymorphisms in FHM genes have not been generally found to be associated with common migraine [79] and references therein. Neurological candidate genes of the serotonergic and dopaminergic systems have predominantly been investigated in migraine, given that it is a neurological disease with autonomic nervous system symptoms. Receptors, transporters and enzymes involved in neurotransmitter synthesis have been targeted. Evidence for the involvement of serotonin stems from biochemical studies in the 1960s which demonstrated altered levels of circulating 5-HT levels in the urine and platelets of migraineurs during their attacks [80]. Also the fact that serotonin and other neurotransmitters can trigger or affect vascular dysfunction/tone stimulated scientific interest in genes of these neurotransmitter systems as targets to investigate. A fine balance in and across neurotransmitter systems is necessary to maintain normal physiological processes and low function in one system can in turn affect the activity of the other in a cumulative manner. 

Various serotonin receptors have been investigated however the most interesting finding was association in the human serotonin transporter *SLC6A4* gene located on chromosome 17q11.2 and migraine [81]. The *SLC6A4 *serotonin transporter codes for an integral membrane protein that clears serotonin at the synapse and recycles it back into neurons and blood platelets. Two main polymorphisms have been identified in this gene and investigated in association studies. One is a 44bp insertion/deletion functional polymorphism in the promoter region, termed 5-HTTLPR with two common allelic forms, the short variant (S) has 14 repeats of a sequence and the long variant (L) has 16 repeats [82]. Initial evidence of association with the promoter 5-HTTLPR polymorphism was correlated with possession of the short S allele [83]. Possession of the S allele results in slower clearing of serotonin from the synaptic cleft due to down-regulation in gene expression meaning that migraineurs express only half the number of serotonin transporters. Some studies identified an association between the S allele in the promoter 5-HTTLPR polymorphism and migraine [84-87], while others found no evidence of association [88, 89]. Schurks *et al.*, 2010 [82] reviewed the literature and performed a meta-analysis of 10 studies to determine if any association existed between the 5-HTTLPR polymorphism and migraine and their results indicate no overall association. 

The second polymorphism investigated in the *SLC6A4 *gene consists of a 17bp variable number of tandem repeats known as (STin2 VNTR) in intron 2 with 2 common alleles STin2.10 and STin2.12 composed of 10 or 12 repeat units [82]. Schurks *et al.*, 2010 [90] conducted a meta-analysis of 5 studies considering this polymorphism and found that 5-HTT VNTR STin2 12/12 genotype is associated with an increased susceptibility to migraine especially among populations of European descent. In an attempt to resolve discrepancies in results of association studies Liu *et al.*, 2011 reviewed 15 studies for meta-analysis and found that the 5-HTT VNTR STin2 12/12 genotype confers an increased risk for migraine in the general population [91]. The two polymorphisms 5-HTTLPR and VNTR in the serotonin transporter *SLC6A4* gene have been associated with slower removal of 5-HT at the synapse. 

The dopaminergic system has also been investigated in migraine because the interaction of Dopamine (DA) with its receptors is known to mediate certain prodromal symptoms experienced by migraineurs. Dopamine receptor antagonists are effective at relieving migraine and DA receptors have been localised in the trigeminovascular system an integral component of migraine pain mechanisms [92]. Also reports in the literature exist that migraineurs have an increased density of dopamine receptors DRD3 and DRD4 on lymphocytes and that DA agonists can bring about migraine [93, 94]. Genetic studies have thus focused on candidate genes encoding proteins of the dopaminergic system, including DA receptors, DA transporter proteins and enzymes involved in the synthesis and metabolism of DA.

Interest in the role of dopamine in migraine was motivated after Peroutka *et al.* 1997 identified a susceptibility polymorphism at rs61689984 in the DRD2 gene with increased frequency of the C allele in migraine with aura (0.84) compared to migraine without aura (0.70) and controls (0.71) [95]. Del Zompo *et al.*, 1998 subsequently analysed a number of candidate genes *DRD2*, *DRD3* and *DRD4* in a Sardinian population but found no association [96]. Todt *et al.* 2009 investigated a total of 53 SNPs in 10 genes from the dopaminergic system, including *COMT, DBH, DDC, DRD1, DRD2, DRD3, DRD4, DRD5 SLC6A3* and *TH*, in a large German case-control cohort of migraine with aura and from this study the dopamine transporter *SLC6A3* 5p15 and *DBH* 9q34 enzyme emerged as significant [97]. *SLC6A3* is an important transporter of dopamine that maintains a low concentration of DA in the extracellular space. However it should be noted that this gene, also named *DAT1*, showed no association in other studies [98-100].

The DBH locus 9q34 encodes the Dopamine Beta Hydroxylase gene an enzyme involved in synthesis of noradrenaline from the substrate dopamine. There have been some reports of elevated serum levels of DBH enzyme in migraineurs during an attack [101]. Polymorphisms that reduce the plasma enzymatic activity of this gene and lead to an increase in DA have been investigated. The functional *DBH* insertion/deletion polymorphism 19bp indel (-4784-4803) was found to associate with migraine in an Australian population [102]. In particular migraine risk increases in males with the homozygous del/del genotype up to three times [102]. Also a promoter functional polymorphism (-1021C→ T, rs1611115) in DBH which reduces plasma enzyme activity by up to 52% was found associated with migraine aura in an Australian population [103]. The level of individual DBH activity has a strong genetic background. The study by Todt *et al.* 2009, showed significant association for another SNP rs2097629 in a German MA population which is not in LD with the promoter polymorphisms [97]. Another group Corominas *et al.* 2009 analysed 50 tag SNPs in 8 genes from the dopaminergic system: *DRD1*, *DRD2*, *DRD3*, *DRD5*, *DBH*, *COMT*, *SLC6A3 *and *TH *in two case control populations of Spanish origin and found no evidence of robust genetic association of any of these genes with migraine. Investigations in dopamine genes have shown some relationship to migraine but results have not always replicated. The *DBH* locus and the *SLC6A3* dopamine transporter seem to correlate with more dopamine at the synapse and support a hypersensitivity to DA hypothesis [104]. 

The glutamatergic system has not been studied as extensively as that of serotonin and dopamine however the biological constituents including enzymes, glutamate receptors and transporters which mediate excitatory signals via ionotropic and metabotropic receptors have been proposed as candidates to investigate in light of recent genetic studies implicating glutamate in migraine [105-107]. Further research into the glutamatergic system is necessary to confirm its role in migraine susceptibility and pathophysiology. 

### 4.2. Vascular Pathways

The link between migraine and the vascular system was encapsulated in the vascular theory proposed by Graham and Wolff in the 20^th^ Century when they hypothesized that pain arises due to dilated blood vessels. Current revised theories of migraine pathophysiology suggest an interaction between cranial blood vessels and the brain’s neural circuitry. The role of the vasculature in migraine pathophysiology is certainly well established given the effects of vasoactive drugs and the observation that migraine is co-morbid with vascular conditions of stroke, Patent Foramen Ovale (PFO) and hypertension [10]. There is a well-known relationship between migraine aura and cardiovascular disease however no causal link has been established [108, 109]. Susceptibility loci on different chromosomes that affect vascular endothelial function have been investigated. These include variants such as the renowned and most cited C677T polymorphism in the homocysteine metabolism gene, *MTHFR *to nitric oxide synthase (NOS), calcitonin gene-related peptide (CGRP), angiotensin I-converting enzyme (ACE), and the NOTCH3 gene.

The NOTCH3 gene is a gene that encodes a transmembrane receptor involved in cellular differentiation and cell cycle regulation [49, 110]. This gene was identified in a subset of families segregating with cerebral autosomal dominant arteriopathy with subcortical infarcts and leukoencephalopathy (CADASIL; MIM125310) [49]. CADASIL is an inherited condition that causes stroke and other impairments. The underlying pathology of CADASIL is progressive loss of vascular smooth muscle cells in the tunica media of small arteries, and accumulation of granular osmiophilic material (GOM) that is detected by electron microscopy [110]. This damage reduces blood flow to various tissues in the body resulting in ischemia. This blood vessel damage can interestingly cause migraine and other impairments of normal brain function. 

Studies of polymorphisms at the *MTHFR* locus are not unique to migraine as it is a gene which has been implicated in a multitude of diseases due to its central role in the homocysteine (Hcy) pathway. Diseases that have detected a correlation with this gene include neural tube defects, heart disease, stroke, high blood pressure (hypertension), high blood pressure during pregnancy (preeclampsia), an eye disorder called glaucoma, psychiatric disorders, and certain types of cancer [111]. Some of these conditions are co-morbid with migraine. The *MTHFR* gene located on chromosome 1p36.3 codes for an important enzyme methylenetetrahydrofolate reductase in the Hcy pathway, a pathway that is pivotal to many functions throughout the body including DNA methylation, immune function, muscle metabolism, and regulation of cardiovascular and central nervous system health and removal of toxins [112]. This enzyme is responsible for reducing 5,10-methylenetetrahydrofolate to active 5-methyltetra- hydrofolate, the substrate required for the conversion of homocysteine to methionine in the Hcy pathway [112]. When the MTHFR protein or its levels are compromised Hcy accumulates in the bloodstream and the production of methionine is reduced. 

This situation can arise because of genetic alterations in *MTHFR*. These have been well characterized with more than 40 different polymorphisms identified in the *MTHFR* gene that reduce the activity of the enzyme and lead to an excess of Hcy in the bloodstream [113]. In migraine the C to T single nucleotide polymorphism (SNP) at codon 677 of the *MTHFR* gene has been the most extensively studied functional polymorphism for a common migraine type. The *MTHFR *C677T variant changes an alanine to a valine within the catalytic domain of the enzyme, affecting the quaternary structure of the protein and reducing its enzymatic activity by up to 50% [114]. This reduced enzyme activity leads to higher levels of homocysteine in the blood which contributes to a condition known as mild hyperhomocysteineimia which is a risk factor for migraine and a number of cardiovascular diseases [4]. Although deficiency in MTHFR functionality can produce mild hHcy in humans, this condition can also occur due to environmental factors such as insufficient dietary intake of folate B_9_, vitamin B_12_, and vitamin B_6_. 

A few studies indicate that excess Hcy in the blood can cause remodelling of vascular tissue via pathological mechanisms that stimulate the growth of smooth muscle cells, cause endothelial dysfunction by down-regulating expression of endothelial NO synthase and release of inflammatory mediators [115-120]. The vascular changes produced by Hcy are detrimental to arteriolar integrity and can make people more susceptible to vascular inflammation and atherogenesis, which in turn can result in ischemic injury. This has been suggested as a mechanism contributing to migraine on a vascular or ischemic basis. 

Although numerous studies have investigated the genetic association of this polymorphism in different migraine populations the status of this gene remains controversial with positive and negative results obtained by independent groups. A couple of Meta analyses have however shed some light on the hypothesised role of the *MTHFR* gene in migraine. Rubino *et al.*, 2009 pooled results from 8 published articles and identified a significant association for the *MTHFR* C677T, TT genotype polymorphism but in MA only [111]. A recent Meta analysis conducted by Shurks *et al.*, 2010 pooling results from 13 studies investigating the association of MTHFR C677T variant and migraine identified a positive association for the TT genotype in the MA only group [121]. This study demonstrates that migraine sufferers in particular MA carry the *MTHFR* 677TT genotype. The TT genotype was associated in both meta-analyses with an increased risk for migraine with aura. It has been suggested that there may be genetic differences which are specific to each of the migraine subtypes. People who have inherited the *MTHFR* TT genotype have a less active enzyme and are said to be genetically slower homocysteine metabolizers. 

The biochemical properties of wild type and mutant MTHFR enzymes reveal that mutations decreasing the activity of MTHFR can contribute to high circulating plasma homocysteine and mild hyperhomocysteineimia, a condition which may contribute to migraine [122]. *MTHFR* knockout mice show elevated levels of plasma and total homocysteine in comparison to wild-type and suffer other abnormalities, including hypomethylation of their DNA, developmental delay, and thromboses of the arteries and cerebral veins [112]. Biochemical and animal studies have been useful in demonstrating that these abnormalities are due to non-functional MTHFR [112]. 

A genetic defect in *MTHFR* activity can be bypassed by increasing dietary intake of B vitamins as shown in a pilot study [65] of migraineurs supplemented with folate B_9 _and vitamins B_12_ and B_6_ over a six month period. Study participants reported an improvement in migraine symptoms, this improved response was correlated with a reduction in homocysteine levels from the supplementation with B vitamins and with carriage of at least one C allele [123]. This study shows that supplementation with B vitamins can help restore balance in this pathway in individuals with this mutation and is an effective Nutrigenomics measure. Clinical trials examining the role of B vitamins in vascular disorders have also implicated *MTHFR* as a culprit of disease and demonstrated that dietary supplementation with B vitamins can reduce the risk of disease [124-130].

### 4.3. Hormonal Pathways

The influence of female sex hormones in migraine is evident from the noticeably distorted gender ratio (3:1 female to male) clearly observed in migraine prevalence studies [131]. Accordingly women are 3 times more likely to suffer migraine than men, a fact attributed to the influence of menstruation, pregnancy and menopause [132, 133]. Genetic studies of hormonal genes have focused on the estrogen receptor (*ESR1*) and the progesterone receptor (*PGR*) genes, a logical choice based on fluctuating hormones of the ovarian cycle which many women recognize as triggers for their migraine.

The observation that migraine prevalence varies with hormonal transitions including pregnancy and menopause has stimulated scientific interest into the impact that endogenous and exogenous hormones have on the frequency and severity of migraine attacks in women. The general trend observed is that migraine affects boys and girls equally at puberty and thereafter increases in adulthood with women predominantly affected [134]. During pregnancy oestrogen is constantly high whilst during menopause hormones fluctuate for a while but then level out and are constantly low post menopause, it is at these times that migraine attacks diminish [135]. Thus lack of hormonal balance, i.e. rapid rise and fall in the circulation is thought to contribute to migraine and may be a trigger particularly in menstruating women as this is the time when hormones are likely to fluctuate [136].

The ovarian hormones estrogen and progesterone are steroid hormones capable of modulating many biological functions in either a genomic (transcription dependent) or nongenomic (non–transcription dependent) mechanism via their cognate receptors [136]. The estrogen and progesterone receptors are among the most studied genes in relation to hormonal pathways in migraine and besides their obvious role in sexual development and reproductive function they also affect functioning of the cardiovascular and nervous systems as well as growth and maintenance of the skeleton [137]. The effect of exogenous estrogen and progesterone in the form of oral contraception or hormone replacement therapy have shown that some womens migraine symptoms can improve because of stabilizing hormonal fluctuations but at the same time can also worsen migraine in certain people [138]. Consequently hormone administration as a therapy in the treatment of migraine is not indicated.

Polymorphisms in *ESR1* and *PGR* have been studied with respect to migraine susceptibility: some studies have shown that independent polymorphisms in *ESR1* and *PGR* are associated with increased migraine risk whilst other studies have detected no association whatsoever. Initial positive findings were detected for *ESR1 *by Colson *et al.*, 2004 [139] for the G594A SNP rs2228480 in exon 8 in two independent Australian case-control populations (population 1, p<0.008, population 2, p<4x10^-5^). Variation in this gene is particularly interesting because estrogen receptors have been implicated in disease processes in breast cancer, endometrial cancer, and osteoporosis and the *ESR1* SNP rs2228480 was previously found to show association with breast cancer [140]. *ESR1* is localized to chromosome 6q25.1 and is expressed in many areas of the brain regulating many functions including regulating gene expression through cell signalling affecting glutamate and serotonin synthesis and CGRP and can regulate vascular tone by stimulating release of NO [141, 142]. 

The association of this SNP 594G>A (exon 8) with migraine however was not replicated in 3 subsequent studies [143-145]. A further 3 SNPs were investigated in *ESR1* namely, 325C>G (exon 4), T/C *Pvu*II SNP (intron 1) and T30C. For SNP 325C>G (exon 4), 5 studies reported no association and 2 studies reported a positive association in a Caucasian population. The T/C *Pvu*II SNP (intron 1) was interrogated in two studies only one reporting a positive association in an Indian population and the T30C was only investigated in a Spanish study and no association detected [146]. In summary the synonymous polymorphisms 325C>G (exon 4) and 594G>A (exon 8) have been investigated the most for *ESR1 *and migraine. Although they are located in exonic regions of the gene their functional implication in disease is unknown and some have indicated they may be in LD with a nearby causal variant yet to be identified. The ethnicity of the populations used in these studies included, Caucasian, Indian, Finnish and Spanish.

Subsequent to the estrogen receptor, the PROGINS variant (a 306 base pair insertion within intron 7) in *PGR* located on chromosome 11q22, was next investigated by Colson *et al.*, 2005 in the same Caucasian population. Colson *et al.*, 2005 found a positive association again only to be replicated by Lee *et al.*, 2007 in patients with migraine-associated vertigo and Joshi *et al.*, 2010 in a north Indian population [147]. Interestingly when the original authors analyzed the interaction of both hormonal genes together (*ESR1* 594A allele and PROGINS variant) they identified a synergistic effect whereby migraine risk was increased 3.2 times [148]. 

Oterino, *et al.*, 2008 approached the relationship of hormone receptors and migraine from a multigenic, gene-gene interactions perspective [149]. They analysed 5 estrogen related genes: oestrogen receptor 1 gene (*αESR1*), oestrogen receptor 2 gene (*βESR2*), follicle stimulating hormone receptor gene (*FSHR*), CYP19 aromatase gene polypeptide A1 (*CYP19A1*) and nuclear receptor interacting protein 1 (*NRIP1*) [149] in case-control cohorts and in pedigrees. The *ESR1* C325G locus was genotyped and the *ESR1* gene emerged as the strongest candidate associated with migraine, a result which is consistent with previous association studies. The other four genes were tested in migraine because they had not been previously included and they are steroid hormone genes which converge in the estrogenic pathway. *FSHR* and *ESR2* showed less significant association in comparison to *ESR1* (p<0.05) higher in the MA phenotype when analysed singularly. Not convinced by this result the authors pursued to analyse the interaction of *ESR2*-*ESR1*-*FSHR* loci together. The best genetic model indicated an interaction in these loci expressed as a risk Haplotype. Two haplotypes were identified only one of which nearly doubled the risk (OR=1.97) for migraine more so in MA and was replicated in family studies. Studying the interaction of genes together is a more powerful approach to understanding the mechanism of gene interactions in disease.

The inconsistencies in association results of hormone receptors were addressed in a review and meta-analysis by Schurks, *et al.*, 2010 [150]. This study included 8 published articles following criteria for reviewing genetic association studies and identified the *ESR1* G594A and C325G SNPs as contributors to migraine with pooled Odds Ratios yielding greater statistical significance. The variants increase migraine risk by 40-60% [150]. In contrast the *PGR* PROGINS variant was not associated. Polymorphisms in *ESR1* and *PGR* may increase migraine prevalence only in some populations demonstrating an ethnic-specific effect. 

In trying to understand the effect of hormones in migraine some researchers have focussed on migraine subtypes occurring around menstruation. The International Headache Society (IHS) now recognizes and has included ‘candidate’ criteria for pure menstrual migraine without aura (PMM) and menstrually related migraine without aura (MRM) in the appendix of their International Classification of Headache Disorders, 3rd Edition [8]. This classification modality specifically requires attacks of migraine to occur within a 5-day menstrual window [151]. In the case of PMM, attacks must occur only around the time of the month in at least two out of three menstrual cycles to establish a pattern that is greater than by chance alone whereas in MRM attacks can additionally occur at other times outside of menstruation [8]. 

Focussing on an “enriched” study group of patients with “menstrual migraine” as highlighted by Colson *et al.*, 2010, whose migraine closely coincides with the hormonal cycle may be the way to go for the future to interpret the relationship between migraine and hormones [152]. Hershey *et al.*, 2012 investigated gene expression in menstrual-related migraine patients (MRM), non-MRM and controls using blood and an affimetrix human exon ST 1.0 array [153]. 279 genes were found differentially expressed for MRM: many of the genes were functionally related having known immune or mitochondrial functions, but only a few were hormone-related. The study supports the notion that although overlaps do exist, MRM patients possess a different genomic expression profile to non MRM individuals, however further validation in other subject groups is required [153]. Although hormones clearly play a role in migraine how they contribute at a physiological level to migraine mechanisms is not well understood and complicated by the complex interactions they have with a number of physiological systems. 

### 4.4. Mitochondrial Pathways

Migraine research has recently taken a new direction by investigating genes of the mitochondrial genome. The evidence supporting the idea of mitochondrial involvement in migraine has accumulated from a variety of biochemical, imaging, morphological and genetic studies. Together the data have raised an interesting hypothesis that a dysfunction of mitochondrial energy metabolism could account for the pathogenesis of some subtypes of migraine. The argument put forth is that decreased energy production brought about by defects in oxidative phosphorylation (OXPHOS) may contribute to an energy imbalance in migraine via a decrease in the threshold for cortical spreading depression. 

OXPHOS is an important metabolic pathway for the production of energy in the form of adenosine tri-phosphate (ATP) that occurs in the mitochondria [154]. Although ATP is normally produced through oxidative phosphorylation it can also be produced via the creatine kinase reaction by transfer of inorganic phosphate (Pi) from phosphocreatine (PCr) to adenosine diphosphate (ADP) [155]. This metabolic shift occurs when mitochondrial enzymes are saturated or during anaerobic conditions and is considered a measure of mitochondrial efficiency [156]. Cells highly dependent on ATP production have the most mitochondria these include neurons and muscle cells and as a result these tissues are the first to be affected in mitochondrial disorders [154]. The brain in particular has a high energy demand and is sensitive to reduced energy production. Pathogenic mutations affecting mitochondrial function can impair energy metabolism and ion homeostasis in neurons resulting in a range of downstream abnormalities, the full extent of which is not yet characterized or fully understood. Certain mitochondrial encephalopathies show clinical resemblance to migraine these include lactic acidosis and stroke-like episodes (MELAS), myoclonic epilepsy with ragged red fibres (MERRF) and Kearns Sayre syndrome and the Leber's hereditary optic neuropathy (LHON) [157]. The majority of these mitochondrial disorders lead to an “energy deficiency” state and some manifest with migraine headache [158].

Morphological and biochemical studies have showed a reduction in the metabolism of cellular energy in different tissues, including brain. Specific morphologic changes in the mitochondria have been detected; these include ragged red fibres (RRFs) with an abnormal number of sarcolemmal mitochondria and cytochrome c oxidase (COX) negative fibres with a higher fat content in the skeletal muscle of some patients with migraine with prolonged aura and FHM patients [159-161]. Biochemical evidence has shown levels of lactic acid in blood and CSF to be higher in migraine patients [162]. Lactic acid in blood is used as a biomarker of metabolic dysfunction and is typically elevated in mitochondrial disorders. Additionally evidence of reduced activity of several mitochondrial enzymes in the mitochondria of platelets of migraineurs supports the involvement of mitochondria in migraine [163-165]. The consequence of these findings has lead to the therapeutic administration of Nutraceuticals acting on mitochondrial metabolism. Magnesium, co-enzyme Q10 and riboflavin which are enzyme co-factors known to affect components of the respiratory chain have shown some promise [166-169]. Additionally supplementation with vitamins B_6_, B_12_ and folate B_9_ have shown a decrease in severity and frequency of MA attacks [123, 170].

More recently non-invasive imaging techniques, such as magnetic resonance spectroscopy (MRS) have been employed in several studies to quantify *in vivo* energy metabolism and cerebral metabolites in brain and muscle of migraine patients [156, 160, 161, 171]. Phosphorus-MRS (P-MRS) measurements of phosphorylated metabolites including Pi, PCr, low-energy phosphates (ADP) and high energy phosphates adenosine triphosphate (ATP) from migraine patients reveal a lower ratio of PCr/Pi correlating with less available energy in neurons [155, 156, 172]. A further marker characteristic of a disturbance in energy metabolism is the accumulation of lactate (Lac) during anaerobic glycolysis due to inefficient pyruvate metabolism resulting in lactic acidosis a condition characterized by low pH in body tissues and blood. Proton-MRS (H-MRS) studies have detected high levels of lactate in patients with migraine [173]. Therefore, MRS studies have revealed a lower energy metabolism in brain and muscle of migraine patients which is possibly linked to mitochondrial defects. These studies hint at a mitochondrial component in migraine however it is unclear at this stage if these differences are due to a primary mitochondrial dysfunction or secondary to alterations in brain excitability and thus further studies utilizing consistent methodology in larger homogenous populations are needed [155]. 

Mutations associated with mitochondrial encephalopathies have been investigated for association in migraine susceptibility however so far no specific mt polymorphisms have been associated with migraine. The most interesting study by Zaki *et al.*, 2009 found two polymorphisms in the mitochondrial genome C16519T and G3010A associated with migraine and cyclic vomiting syndrome CVS patients [174]. Only a small number of genetic studies have examined the influence of mitochondrial DNA variants in migraine susceptibility and to date little has been concluded from these studies due to small sample size [175-180]. A significant drawback of investigating mitochondrial variants in the population is in the recruitment of sufficient numbers of affected people and also the fact that they are often rare with low allele frequency makes it difficult to obtain statistically meaningful results. To overcome some of these obstacles it may be more fruitful to invest in full mitochondrial genome sequencing rather than funding small scale underpowered projects. In summary a thorough examination of mtDNA and nuclear mitochondrial genes and epistatic interactions between the mitochondrial and nuclear genomes would help to address the question of whether a mitochondrial mechanism in migraine is at play.

### 4.5. Migraine Genome Wide Association Studies

Genome wide association studies (GWAS) allow scanning of the genome for gene regions associated with a disease without *a priori* assumptions about disease aetiology. GWAS has emerged as a powerful genotyping technique capable of genotyping millions of SNPs across the entire genome and involves screening for potential associations between SNPs and complex diseases using DNA from people affected with a disease (cases) and healthy individuals (controls). GWAS studies have been possible thanks to the completion of the Human Genome Project, International HapMap Project, the development of dense genotyping chips and the availability of biobanks, repositories of human genetic material [181]. A GWAS is conducted using SNP arrays, the most popular platforms currently being Illumina and Affimetrix [182]. 

The first migraine GWAS was of European migraineurs [183] and identified one SNP that reached genome wide significance, this was susceptibility variant rs1835740 at locus 8q22.1 [183] (Table **2**). This marker rs1835740 is located between the *PGCP* and *MTDH* genes, both of which affect the accumulation of glutamate at the synapse. The authors tested the marker’s effect on gene expression by using fibroblasts, primary T cells, and LCLs from umbilical cords in an attempt to propose a mechanism to explain its role in migraine. They showed the risk allele A of this marker to be associated with higher levels of expression of *MTDH*. *MTDH* in turn down-regulates *EAAT2* gene: a protein responsible for removing glutamate from synapses in the brain. Consequently rs1835740 may contribute to migraine through its effect on *MTDH* and *EAAT2*. Reduced activity of *EAAT2* may lead to too much extracellular glutamate which may increase susceptibility to CSD. 

The second migraine GWAS conducted a meta-analysis of six European cohorts. The most interesting finding from this study is the SNP rs9908234 in the nerve growth factor receptor (NGFR) gene with (P-value 8.00x10^-8^). The association of this SNP was not replicated in 3 cohorts in the Netherlands and Australia suggesting that population genetics is more complex than expected and hence the need for replication cohorts to validate potential associations [184]. The reason provided by the authors for lack of replication of this SNP is insufficient power due to the nature of the population-based cohorts employed. The cohort used in the study was heterogeneous consisting of patients with variable severity of migraine and a smaller genetic component which the authors argue may prohibit replication. This study also identified modest support for association with the *MTDH* gene (astrocyte elevated gene 1 or AEG-1) previously associated in the Antilla, *et al.*, 2010 study. 

The third GWAS was conducted in a cohort of European women from the Women’s Genome Health Study (WGHS) including 5,122 migraineurs and 18,108 controls [185]. This is the largest migraine GWAS undertaken with a sample size of 23,230. Three new loci were identified, rs2651899 (1p36.32, *PRDM16*), rs10166942 (23q37.1, *TRPM8*) rs11172113 (12q13.3, *LRP1*) [185]. Little is known about the function of PRDM16 and how it relates to migraine. *TRPM8* belongs to the TRP super family of channels activated by chemo- and somato-sensation involved in pain pathways. *LRP1* is a lipoprotein expressed in brain and vasculature that is co-localized with glutamate receptors in neurons and lends some support to the involvement of the neurotransmitter glutamate in migraine pathophysiology. In conclusion this study has identified 3 unique SNPs in different pathways, although how they each contribute to migraine remains to be determined.

The most recent GWAS was conducted in people of German and Dutch origin using the migraine without aura phenotype only [186]. The previously identified loci *TRPM8* and *LRP1* were replicated in this GWAS and two new loci *MEF2D* (myocyte enhancer factor 2D), a transcription factor that regulates neuronal differentiation and *TGFBR2* (encoding transforming growth factor β receptor 2) were identified. Minor associations in two other genes *PHACTR* (phosphatase and actin regulator 1) and *ASTN2* were also reported. However replication and functional studies are needed to better understand the involvement and significance of these genes in migraine aetiology. 

Although the contribution of GWAS findings to current knowledge of migraine has been modest with a total of four genomic regions found to be significantly and reproducibly implicated, further studies with larger sample groups will increase the power and help to reveal further genes that contribute to migraine. Importantly the genes that have been identified via GWAS with respect to migraine to date are novel and offer new avenues to pursue. While GWAS is effective at identifying novel genes or genomic regions associated with a disease phenotype, it does not identify the causal variant involved at the detected locus or address gene function. However, GWAS can provide fresh insight from which to study how the genes function and contribute to the phenotype or a disease pathway. 

## CONCLUSIONS AND FUTURE DIRECTIONS

Migraine is a well-studied disorder that results from the interaction of multiple genes with environmental triggers. Physiological studies using new and non-invasive imaging techniques have led to a clearer understanding of migraine pathophysiology providing insights into the basic functioning of the neuronal and vascular systems and how they interact to produce the full spectrum of migraine symptoms. However the origin of the pain experienced during migraine episodes is still widely debated. Similarly genetic studies have produced a large list of genes implicated in migraine, but how they actually participate in migraine processes is still poorly understood for the majority of them leaving many gaps in the jigsaw to be filled. High throughput genotyping and next generation sequencing techniques will greatly enhance the study of migraine genetics in the near future. In particular exome and full genomic sequencing will make detecting mutations involved in migraine that show strong familial inheritance an attainable goal. Future GWAS conducted with larger numbers of samples, will most likely lead to the discovery of more genes of small effect that potentially contribute to the risk of developing migraine and identify genes specific to migraine subgroups, such as MO, MA and MRM. Although there are some effective anti-migraine therapies available and more being developed, their mechanisms of action are not completely known and it is therefore not understood why they do not work for some individuals. More complete understanding of the molecular pathways involved and the relevant genomic profile of migraineurs will aid in the development of new anti-migraine drugs and treatments, and or enable those currently available to be better targeted to suit individuals. Finally, because migraine is a complex disorder with no identifiable pathology the best way to move forward is with a multidisciplinary approach incorporating results from emerging imaging techniques, biochemical, pharmacologic and genetic studies in order to better understand the molecular basis of this debilitating disease.

## Figures and Tables

**Table 1.** Familial Hemiplegic Migraine and Defective Genes Identified

	Gene	Chromosome Location	Protein
FHM1 (MIM141500)	*CACNA1A*	19p13	Pore-forming α1 subunit of neuronal Ca2.1(P/Q type) voltage-gated calcium channels
FHM2 (MIM609634 )	*ATP1A2*	1q23	Catalytic α2 subunit of a glial and neuronal sodium-potassium pump
FHM3 (MIM602481)	*SCN1A*	2q24	Pore forming α1 subunit of neuronal Na1.1 voltage-gated sodium channels

**Table 2.** Migraine GWAS Studies

Reference	Variants Identified	CASES/CONTROLS	Origin of Samples	Replication Samples
				P value OR (95% CI)
Anttila *et al.* 2010	rs1835740 (between MTDH and PGCP)	2,731/10,747	Clinic-based; Finland, Germany, The Netherlands	1.69x10-11, OR 1.18, (1.13-1.24)
Ligthart *et al.* 2011	rs9908234 (NGFR)	2,446/8,534	Population-based; The Netherlands, Iceland	8.00x 10-8
Chasman *et al.* 2011	rs2651899 (PRDM16) rs10166942 (TRPM8) rs11172113 (LRP1)	5,122/18,108	Population-based; US, European descent	3.8x10-9, OR 1.11 (1.07-1.15) 5.5x10-12, OR 0.85 (0.82-0.89) 4.3x10-9, OR 0.90 (0.87-0.93)
Freilinger *et al.* 2012	MEF2D Rs1050316 Rs 2274316 Rs 1925950 Rs 3790455 Rs 3790459 Rs 12136856 TGFBR2 Rs 7640543 PHACTR1 Rs 9349379 ASTN2 Rs 6478241 TRPM8 Rs 10166942 Rs 17862920 LRP1 Rs 11172113	2,326/4,580	German-Dutch individuals	7.06x10-11, OR 1.2 (1.14-1.27) 1.17x10-9, OR 1.19 (1.13-1.26) 3.20x10-8, OR 0.86 (0.81-0.91) 3.86x10-8 1, OR 1.16 (1.10-1.23) 9.83x10-13, OR 0.78 (0.73-0.84) 2.97x10-8, OR 0.86 (0.81-0.91)

## References

[1] Stovner LJ, Hagen K (2006). Prevalence burden and cost of headache disorders. Curr. Opin. Neurol.

[2] Bigal ME, Lipton RB (2009). The epidemiology burden and comorbidities of migraine. Neurol. Clin.

[3] Stovner L, Hagen K, Jensen R, Katsarava Z, Lipton R, Scher A, Steiner T, Zwart JA (2007). The global burden of headache: a documentation of headache prevalence and disability worldwide. Cephalalgia.

[4] Stovner LJ (2006). Headache epidemiology how and why. J Head Pain.

[5] Wessman M, Kaunisto MA, Kallela M, Palotie A (2004). The molecular genetics of migraine. Ann. Med.

[6] Montagna P (2008). Migraine a genetic disease. Neurol. Sci.

[7] Mueller LL (2007). Diagnosing and managing migraine headache. J. Am Osteopath Assoc.

[8] Zafar S (2013). Headache Classification Committee of the International Headache Society The International Classification of Headache Disorders 3rd edition (beta version). Cephalalgia.

[9] Viana M, Sprenger T, Andelova M, Goadsby PJ (2013). The typical duration of migraine aura: a systematic review. Cephalalgia.

[10] Dalkara T, Nozari A, Moskowitz MA (2010). Migraine aura pathophysiology the role of blood vessels and microembolisation. Lan. Neurol.

[11] Villalon CM, Centurion D, Valdivia LF, deVries P, Saxena PR (2003). Migraine pathophysiology pharmacology treatment and future trends. Curr. Vasc. Pharmacol.

[12] Tajti J, Pardutz A, Vamos E, Tuka B, Kuris A, Bohar Z, Fejes A, Toldi J, Vecsei L (2011). Migraine is a neuronal disease. J. Neural Transm.

[13] Dalkara T, Zervas NT, Moskowitz MA (2006). From spreading depression to the trigeminovascular system. Neurol. Sci.

[14] Goadsby PJ, Charbit AR, Andreou AP, Akerman S, Holland PR (2009). Neurobiology of migraine. Neuroscience.

[15] Levy D (2010). Migraine pain and nociceptor activation where do we stand. Headache.

[16] Rapoport A, Edmeads J (2000). Migraine The evolution of our knowledge. Arch. Neurol.

[17] Eadie MJ (2005). The pathogenesis of migraine 17th to early 20th Century understandings. J. Clin. Neurosci.

[18] Baron EP, Tepper SJ (2010). Revisiting the role of ergots in the treatment of migraine and headache. Headache.

[19] Spierings EL (2009). Migraine migraine headache pathogenesis in historical perspective. Rev. Neuro. Dis.

[20] Humphrey PP (2008). The discovery and development of the triptans a major therapeutic breakthrough. Headache.

[21] Amin FM, Asghar MS, Hougaard A, Hansen AE, Larsen VA, deKoning PJH, Larsson HBW, Olesen J, Ashina M (2013). Magnetic resonance angiography of intracranial and extracranial arteries in patients with spontaneous migraine without aura a cross-sectional study. Lan. Neurol.

[22] Edvinsson L, Villalon CM, MaassenVanDenBrink A (2012). Basic mechanisms of migraine and its acute treatment. Pharmacol. Ther.

[23] Parsons AA, Strijbos PJ (2003). The neuronal versus vascular hypothesis of migraine and cortical spreading depression. Curr. Opin. Pharmacol.

[24] Moskowitz MA (2008). Molecular mechanism of migraine. Rinsho Shinkeigaku.

[25] May A, Goadsby PJ (1999). The trigeminovascular system in humans.Pathophysiologic implications for primary headache syndromes of the neural influences on the cerebral circulation. J. Cereb. Blood Flow Metab.

[26] Arulmani U, Maassenvandenbrink A, Villalon CM, Saxena PR (2004). Calcitonin gene-related peptide and its role in migraine pathophysiology. Eur. J. Pharmacol.

[27] Samsam M, Covenas R, Ahangari R, YajeyaJavier J (2010). Major Neuroanatomical and Neurochemical Substrates Involved in Primary Headaches.

[28] Moskowitz MA (2008). Defining a pathway to discovery from bench to bedside. The trigeminovascular system and sensitization. Headache.

[29] Teive HA, Kowacs PA, MaranhaoFilho P, Piovesan EJ, Werneck LC (2005). Leao's cortical spreading depression: from experimental "artifact" to physiological principle. Neurology.

[30] Hadjikhani N, SanchezDelRio M, Wu O, Schwartz D, Bakker D, Fischl B, Kwong KK, Cutrer FM, Rosen BR, Tootell RB, Sorensen AG, Moskowitz MA (2001). Mechanisms of migraine aura revealed by functional MRI in human visual cortex. Proc. Natl. Acad. Sci. USA.

[31] Borsook D, Hargreaves R (2010). Brain imaging in migraine research. Headache.

[32] Sprenger T, Borsook D (2012). Migraine changes the brain: neuroimaging makes its mark. Curr. Opin. Neurol.

[33] Moskowitz MA (2007). Genes proteases cortical spreading depression and migraine impact on pathophysiology and treatment. Funct. Neurol.

[34] Eikermann-Haerter K, Moskowitz MA (2008). Animal models of migraine headache and aura. Curr. Opin. Neurol.

[35] Bolay H, Reuter U, Dunn AK, Huang Z, Boas DA, Moskowitz MA (2002). Intrinsic brain activity triggers trigeminal meningeal afferents in a migraine model. Nat. Med.

[36] Karatas H, Erdener SE, Gursoy-Ozdemir Y, Lule S, Eren-Kocak E, Sen ZD, Dalkara T (2013). Spreading Depression Triggers Headache by Activating Neuronal Panx1 Channels. Science.

[37] Bergerot A, Holland PR, Akerman S, Bartsch T, Ahn AH, MaassenVanDenBrink A, Reuter U, Tassorelli C, Schoenen J, Mitsikostas D D, vandenMaagdenberg AMJM, Goadsby PJ (2006). Animal models of migraine looking at the component parts of a complex disorder. Eur. J. Neuro-sci.

[38] Andreou AP, Summ O, Charbit AR, Romero-Reyes M, Goadsby PJ (2010). Animal models of headache: from bedside to bench and back to bedside. Exp. Rev. of Neurother.

[39] James MF, Smith MI, Bockhorst KH, Hall LD, Houston GC, Papadakis NG, Smith JM, Williams AJ, Xing D, Parsons AA, Huang CL, Carpenter TA (1999). Cortical spreading depression in the gyrencephalic feline brain studied by magnetic resonance imaging. J. Physiol.

[40] Fabricius M, Fuhr S, Bhatia R, Boutelle M, Hashemi P, Strong AJ, Lauritzen M (2006). Cortical spreading depression and peri-infarct depolarization in acutely injured human cerebral cortex. Brain.

[41] Olesen J, Jansen-Olesen I (2012). Towards a reliable animal model of migraine. Cephalalgia.

[42] Melo-Carrillo A, Lopez-Avila A (2013). A chronic animal model of migraine induced by repeated meningeal nociception characterized by a behavioral and pharmacological approach. Cephalalgia.

[43] López-Avila A, Melo A, Simón-Arceo K, Coffeen U, Lamothe-Molina P, Ortega-Legaspi JM, Pellicer F, Castillo-Tovar A, Jaimes O (2009). 374 A chronic animal model for the study of migraine. Eur. J. Pain.

[44] Ophoff RA, Terwindt GM, Vergouwe MN, vanEijk R, Oefner PJ, Hoffman SM, Lamerdin JE, Mohrenweiser HW, Bulman DE, Ferrari M, Haan J, Lindhout D, vanOmmen GJ, Hofker MH, Ferrari MD, Frants RR (1996). Familial hemiplegic migraine and episodic ataxia type-2 are caused by mutations in the Ca2+ channel gene CACNL1A4. Cell.

[45] Carrera P, Stenirri S, Ferrari M, Battistini S (2001). Familial hemiplegic migraine: a ion channel disorder. Brain Res. Bull.

[46] Thomsen LL, Kirchmann M, Bjornsson A, Stefansson H, Jensen RM, Fasquel AC, Petursson H, Stefansson M, Frigge ML, Kong A, Gulcher J, Stefansson K, Olesen J (2007). The genetic spectrum of a population-based sample of familial hemiplegic migraine. Brain.

[47] Dichgans M, Freilinger T, Eckstein G, Babini E, Lorenz-Depiereux B, Biskup S, Ferrari MD, Herzog J, vandenMaagdenberg AMJM, Pusch M, Strom TM (2005). Mutation in the neuronal voltage-gated sodium channel SCN1A in familial hemiplegic migraine. Lancet.

[48] DeFusco M, Marconi R, Silvestri L, Atorino L, Rampoldi L, Morgante L, Ballabio A, Aridon P, Casari G (2003). Haploinsufficiency of ATP1A2 encoding the Na+/K+ pump alpha 2 subunit associated with familial hemiplegic migraine type 2. Nat. Gen.

[49] Liem MK, Oberstein SA, vanderGrond J, Ferrari MD, Haan J (2010). CADASIL and migraine: A narrative review. Cephalalgia.

[50] Pietrobon D (2010). CaV2. channelopathies. Pflugers Arch.

[51] Diriong S, Lory P, Williams ME, Ellis SB, Harpold MM, Taviaux S (1995). Chromosomal localization of the human genes for alpha 1A alpha 1B and alpha 1E voltage-dependent Ca2+ channel subunits. Genomics.

[52] Russell MB, Ducros A (2011). Sporadic and familial hemiplegic migraine pathophysiological mechanisms clinical characteristics diagnosis and management. Lan. Neurol.

[53] Dichgans M, Freilinger T, Eckstein G, Babini E, Lorenz-Depiereux B, Biskup S, Ferrari MD, Herzog J, vandenMaagdenberg AM, Pusch M, Strom TM (2005). Mutation in the neuronal voltage-gated sodium channel SCN1A in familial hemiplegic migraine. Lancet.

[54] Meisler MH, Kearney JA (2005). Sodium channel mutations in epilepsy and other neurological disorders. J. Clin. Investig.

[55] Mulley JC, Scheffer IE, Petrou S, Dibbens LM, Berkovic SF, Harkin LA (2005). SCN1A mutations and epilepsy. Hum. Mutat.

[56] deVries B, Stam AH, Kirkpatrick M, Vanmolkot KR, Koenderink JB, vandenHeuvel JJ, Stunnenberg B, Goudie D, Shetty J, Jain V, vanVark J, Terwindt GM, Frants RR, Haan J, vandenMaagdenberg AM, Ferrari MD (2009). Familial hemiplegic migraine is associated with febrile seizures in an FHM2 family with a novel de novo ATP1A2 mutation. Epilepsia.

[57] Kahlig KM, Rhodes TH, Pusch M, Freilinger T, Pereira-Monteiro JM, Ferrari MD, vandenMaagdenberg AM, Dichgans M, George ALJr (2008). Divergent sodium channel defects in familial hemiplegic migraine. Proc. Natl. Acad. Sci. USA.

[58] Garza-Lopez E, Sandoval A, Gonzalez-Ramirez R, Gandini MA, VandenMaagdenberg A, DeWaard M, Felix R (2012). Familial hemiplegic migraine type 1 mutations W1684R and V1696I alter G protein-mediated regulation of Ca(V)2. voltage-gated calcium channels. Biochimica Et Biophysica Acta-Molecular Basis of Disease.

[59] vandenMaagdenberg AMJM, Pietrobon D, Pizzorusso T, Kaja S, Broos LAM, Cesetti T, vandeVen RCG, Tottene A, vanderKaa J, Plomp JJ, Frants RR, Ferrari MD (2004). A Cacna1a Knockin Migraine Mouse Model with Increased Susceptibility to Cortical Spreading Depression. Neuron.

[60] Tottene A, Conti R, Fabbro A, Vecchia D, Shapovalova M, Santello M, vandenMaagdenberg AMJM, Ferrari MD, Pietrobon D (2009). Enhanced Excitatory Transmission at Cortical Synapses as the Basis for Facilitated Spreading Depression in Ca(v)2. Knockin Migraine Mice. Neuron.

[61] Kaja S, VandeVen RCG, Broos LAM, Frants RR, Ferrari MD, VandenMaagdenberg AMJM, Plomp JJ (2010). Severe and Progressive Neurotransmitter Release Aberrations in Familial Hemiplegic Migraine Type 1 Cacna1a S218L Knock-in Mice. J. Neurophysiol.

[62] vandenMaagdenberg AMJM, Pizzorusso T, Kaja S, Terpolilli N, Shapovalova M, Hoebeek FE, Barrett CF, Gherardini L, vandeVen RCG, Todorov B, Broos LAM, Tottene A, Gao Z, Fodor M, DeZeeuw CI, Frants RR, Plesnila N, Plomp JJ, Pietrobon D, Ferrari MD (2010). High Cortical Spreading Depression Susceptibility and Migraine-Associated Symptoms in Ca(V)2. S218L Mice. Ann. Neurol.

[63] Pietrobon D (2005). Function and dysfunction of synaptic calcium channels insights from mouse models. Curr. Opin. Neurobiol.

[64] Leo L, Gherardini L, Barone V, DeFusco M, Pietrobon D, Pizzorusso T, Casari G (2011). Increased susceptibility to cortical spreading depression in the mouse model of familial hemiplegic migraine type 2. PLoS Genet.

[65] Stewart WF, Bigal ME, Kolodner K, Dowson A, Liberman JN, Lipton RB (2006). Familial risk of migraine variation by proband age at onset and headache severity. Neurology.

[66] Mulder EJ, VanBaal C, Gaist D, Kallela M, Kaprio J, Svensson DA, Nyholt DR, Martin NG, MacGregor AJ, Cherkas LF, Boomsma DI, Palotie A (2003). Genetic and environmental influences on migraine a twin study across six countries. Twin Res.

[67] Anttila V, Kallela M, Oswell G, Kaunisto MA, Nyholt DR, Hamalainen E, Havanka H, Ilmavirta M, Terwilliger J, Sobel E, Peltonen L, Kaprio J, Farkkila M, Wessman M, Palotie A (2006). Trait components provide tools to dissect the genetic susceptibility of migraine. Am. J. Hum. Gen.

[68] Nyholt DR, Gillespie NG, Heath AC, Merikangas KR, Duffy DL, Martin NG (2004). Latent class and genetic analysis does not support migraine with aura and migraine without aura as separate entities. Genet. Epidemiol.

[69] Nyholt DR, Morley KI, Ferreira MA, Medland SE, Boomsma DI, Heath AC, Merikangas KR, Montgomery GW, Martin NG (2005). Genomewide significant linkage to migrainous headache on chromosome 5q21. Am. J. Hum. Gen.

[70] Lea RA, Nyholt DR, Curtain RP, Ovcaric M, Sciascia R, Bellis C, Macmillan J, Quinlan S, Gibson RA, McCarthy LC, Riley JH, Smithies YJ, Kinrade S, Griffiths LR (2005). A genome-wide scan provides evidence for loci influencing a severe heritable form of common migraine. Neurogenetics.

[71] Wessman M, Kallela M, Kaunisto MA, Marttila P, Sobel E, Hartiala J, Oswell G, Leal SM, Papp JC, Hamalainen E, Broas P, Joslyn G, Hovatta I, Hiekkalinna T, Kaprio J, Ott J, Cantor RM, Zwart JA, Ilmavirta M, Havanka H, Farkkila M, Peltonen L, Palotie A (2002). A susceptibility locus for migraine with aura on chromosome 4q24. Am. J. Hum. Gen.

[72] Bjornsson A, Gudmundsson G, Gudfinnsson E, Hrafnsdottir M, Benedikz J, Skuladottir S, Kristjansson K, Frigge ML, Kong A, Stefansson K, Gulcher JR (2003). Localization of a gene for migraine without aura to chromosome 4q21. Am. J. Hum. Gen.

[73] Oedegaard KJ, Greenwood TA, Lunde A, Fasmer OB, Akiskal HS, Kelsoe JR (2010). A genome-wide linkage study of bipolar disorder and co-morbid migraine. Replication of migraine linkage on chromosome 4q24 and suggestion of an overlapping susceptibility region for both disorders on chromosome 20p11. J. Affect. Disord.

[74] Lafreniere RG, Cader MZ, Poulin JF, Andres-Enguix I, Simoneau M, Gupta N, Boisvert K, Lafreniere F, McLaughlan S, Dube MP, Marcinkiewicz MM, Ramagopalan S, Ansorge O, Brais B, Sequeiros J, Pereira-Monteiro JM, Griffiths LR, Tucker SJ, Ebers G, Rouleau GA (2010). A dominant-negative mutation in the TRESK potassium channel is linked to familial migraine with aura. Nat. Med.

[75] Lafreniere RG, Cader MZ, Poulin JF, Andres-Enguix I, Simoneau M, Gupta N, Boisvert K, Lafreniere F, McLaughlan S, Dube MP, Marcinkiewicz MM, Ramagopalan S, Ansorge O, Brais B, Sequeiros J, Pereira-Monteiro JM, Griffiths LR, Tucker SJ, Ebers G, Rouleau GA (2010). A dominant-negative mutation in the TRESK potassium channel is linked to familial migraine with aura. Nat. Med.

[76] Wood H (2010). Migraine Familial migraine with aura is associated with a mutation in the TRESK potassium channel. Nat. Rev. Neurol.

[77] King RA, Rotter JIGMA (2002). The Genetic Basis of Common Diseases. Oxford University Press. Oxford.

[78] D'Onofrio M, Ambrosini A, DiMambro A, Arisi I, Santorelli FM, Grieco GS, Nicoletti F, Nappi G, Pierelli F, Schoenen J, Buzzi MG (2009). The interplay of two single nucleotide polymorphisms in the CACNA1A gene may contribute to migraine susceptibility. Neurosci. Lett.

[79] Maher BH, Griffiths LR (2011). Identification of molecular genetic factors that influence migraine. Mol. Genet. Genomics.

[80] Sicuteri F, Anselmi B, Testi A (1961). Biochemical Investigations in Headache. Increase in Hydroxyindoleactic Acid Excretion During Migraine Attacks. Int. Arch. Allergy Appl. Immunol.

[81] Hamel E (2007). Serotonin and migraine biology and clinical implications. Cephalalgia.

[82] Schurks M, Rist PM, Kurth T (2010). 5-HTTLPR polymorphism in the serotonin transporter gene and migraine a systematic review and meta-analysis. Cephalalgia.

[83] Ogilvie AD, Russell MB, Dhall P, Battersby S, Ulrich V, Smith CA, Goodwin GM, Harmar AJ, Olesen J (1998). Altered allelic distributions of the serotonin transporter gene in migraine without aura and migraine with aura. Cephalalgia.

[84] Yilmaz M, Erdal ME, Herken H, Cataloluk O, Barlas O, Bayazit YA (2001). Significance of serotonin transporter gene polymorphism in migraine. J. Neurol. Sci.

[85] Juhasz G, Zsombok T, Laszik A, Gonda X, Sotonyi P, Faludi G, Bagdy G (2003). Association analysis of 5-HTTLPR variants 5-HT2a receptor gene 102T/C polymorphism and migraine. J. Neurogenet.

[86] Borroni B, Brambilla C, Liberini P, Rao R, Archetti S, Gipponi S, Volta GD, Padovani A (2005). Functional serotonin 5-HTTLPR polymorphism is a risk factor for migraine with aura. J. Head. Pain.

[87] Marziniak M, Mossner R, Schmitt A, Lesch KP, Sommer C (2005). A functional serotonin transporter gene polymorphism is associated with migraine with aura. Neurology.

[88] Todt U, Freudenberg J, Goebel I, Heinze A, Heinze-Kuhn K, Rietschel M, Gobel H, Kubisch C (2006). Variation of the serotonin transporter gene SLC6A4 in the susceptibility to migraine with aura. Neurology.

[89] Corominas R, Sobrido MJ, Ribases M, Cuenca-Leon E, Blanco-Arias P, Narberhaus B, Roig M, Leira R, Lopez-Gonzalez J, Macaya A, Cormand B (2010). Association Study of the Serotoninergic System in Migraine in the Spanish Population. Am. J. Med.Gen. Part B-Neuropsyc. Gen.

[90] Schurks M, Rist PM, Kurth T (2010). STin2 VNTR polymorphism in the serotonin transporter gene and migraine: pooled and meta-analyses. J. Head. Pain.

[91] Liu H, Liu M, Wang Y, Wang XM, Qiu Y, Long JF, Zhang SP (2011). Association of 5-HTT gene polymorphisms with migraine a systematic review and meta-analysis. J. Neurol. Sci.

[92] Bergerot A, Storer RJ, Goadsby PJ (2007). Dopamine inhibits trigeminovascular transmission in the rat. Ann. Neurol.

[93] Barbanti P, Fabbrini G, Ricci A, Pascali MP, Bronzetti E, Amenta F, Lenzi GL, Cerbo R (2000). Migraine patients show an increased density of dopamine D3 and D4 receptors on lymphocytes. Cephalalgia.

[94] Barbanti P, Bronzetti E, Ricci A, Cerbo R, Fabbrini G, Buzzi MG, Amenta F, Lenzi GL (1996). Increased density of dopamine D-5 receptor in peripheral blood lymphocytes of migraineurs. A masker for migraine. Neurosci. Lett.

[95] Peroutka SJ, Wilhoit T, Jones K (1997). Clinical susceptibility to migraine with aura is modified by dopamine D2 receptor (DRD2) NcoI alleles. Neurology.

[96] DelZompo M, Cherchi A, Palmas MA, Ponti M, Bocchetta A, Gessa GL, Piccardi MP (1998). Association between dopamine receptor genes and migraine without aura in a Sardinian sample. Neurology.

[97] Todt U, Netzer C, Toliat M, Heinze A, Goebel I, Nurnberg P, Gobel H, Freudenberg J, Kubisch C (2009). New genetic evidence for involvement of the dopamine system in migraine with aura. Hum. Genet.

[98] Karwautz A, CamposdeSousa S, Konrad A, Zesch HE, Wagner G, Zormann A, Wanner C, Breen G, Ray M, Kienbacher C, Natriashvili S, Collier DA, Wober C, Wober-Bingol C (2008). Family-based association analysis of functional VNTR polymorphisms in the dopamine transporter gene in migraine with and without aura. J. Neural. Transm.

[99] McCallum LK, Fernandez F, Quinlan S, Macartney DP, Lea RA, Griffiths LR (2007). Association study of a functional variant in intron 8 of the dopamine transporter gene and migraine susceptibility. Eur. J. Neurol.

[100] Mochi M, Cevoli S, Cortelli P, Pierangeli G, Soriani S, Scapoli C, Montagna P (2003). A genetic association study of migraine with dopamine receptor 4 dopamine transporter and dopamine-beta-hydroxylase genes. Neurol. Sci.

[101] Weinshilboum RM (1978). Serum dopamine beta-hydroxylase. Pharmacol. Rev.

[102] Fernandez F, Lea RA, Colson NJ, Bellis C, Quinlan S, Griffiths LR (2006). Association between a 19 bp deletion polymorphism at the dopamine beta-hydroxylase (DBH) locus and migraine with aura. J. Neurol. Sci.

[103] Fernandez F, Colson N, Quinlan S, MacMillan J, Lea RA, Griffiths LR (2009). Association between migraine and a functional polymorphism at the dopamine beta-hydroxylase locus. Neurogenetics.

[104] Charbit AR, Akerman S, Goadsby PJ (2010). Dopamine what's new in migraine. Cur. Opin. Neurol.

[105] McKenna MC (2007). The glutamate-glutamine cycle is not stoichiometric. Fates of glutamate in brain. J. Neurosci. Res.

[106] Maher BH, Lea R, Follett J, Cox HC, Fernandez F, Esposito T, Gianfrancesco F, Haupt LM, Griffiths LR (2013). Association of a GRIA3 gene polymorphism with migraine in an Australian case-control cohort. Headache J. Head. Face Pain.

[107] Formicola D, Aloia A, Sampaolo S, Farina O, Diodato D, Griffiths LR, Gianfrancesco F, DiIorio G, Esposito T (2010). Common variants in the regulative regions of GRIA1 and GRIA3 receptor genes are associated with migraine susceptibility. BMC Med. Gen.

[108] Schurks M, Buring JE, Kurth T (2010). Migraine migraine features and cardiovascular disease. Headache J. Head. Face Pain.

[109] Pierangeli G, Giannini G, Favoni V, Sambati L, Cevoli S, Cortelli P (2012). Migraine and cardiovascular diseases. Neurol. Sci.

[110] Mosca L, Marazzi R, Ciccone A, Santilli I, Bersano A, Sansone V, Grosso E, Mandrile G, Giachino DF, Adobbati L, Corengia E, Agostoni E, Fiumani A, Gallone S, Scarpini E, Guidotti M, Sterzi R, Ajmone C, Marocchi A, Penco S (2011). NOTCH3 gene mutations in subjects clinically suspected of CADASIL. J. Neurol. Sci.

[111] Rubino E, Ferrero M, Rainero I, Binello E, Vaula G, Pinessi L (2009). Association of the C677T polymorphism in the MTHFR gene with migraine a meta-analysis. Cephalalgia.

[112] Matthews RG (2002). Methylenetetrahydrofolate reductase a common human polymorphism and its biochemical implications. Chem. Rec.

[113] Garilli B (2012). MTHFR Mutation A Missing Piece in the Chronic Disease Puzzle. Summer.

[114] Frosst P, Blom HJ, Milos R, Goyette P, Sheppard CA, Matthews RG, Boers GJ, denHeijer M, Kluijtmans LA, vandenHeuvel LP (1995). A candidate genetic risk factor for vascular disease a common mutation in methylenetetrahydrofolate reductase. Nat. Gen.

[115] Neves MF, Endemann D, Amiri F, Virdis A, Pu Q, Rozen R, Schiffrin EL (2004). Small artery mechanics in hyperhomocysteinemic mice effects of angiotensin II. J. Hypertens.

[116] Smilde TJ, vandenBerkmortel F, Boers GHJ, Wollersheim H, deBoo T, vanLangen H, Stalenhoef AFH (1998). Carotid and femoral artery wall thickness and stiffness in patients at risk for cardiovascular disease with special emphasis on hyperhomocysteinemia. Arterioscl. Throm. Vasc. Biol.

[117] Voutilainen S, Alfthan G, Nyyssonen K, Salonen R, Salonen JT (1998). Association between elevated plasma total homocysteine and increased common carotid artery wall thickness. Ann. Med.

[118] Juo SHH, Liao YC, Kuo CL, Wang Y, Huang CS, Chiang HC, Liu CS (2008). The MTHFR 677 C/T polymorphism influences plasma levels of adhesion molecules and nitric oxide. Thromb. Res.

[119] Chiang JK, Sung ML, Yu HR, Chang HI, Kuo HC, Tsai TC, Yen CK, Chen CN (2011). Homocysteine Induces Smooth Muscle Cell Proliferation Through Differential Regulation of Cyclins A and D1 Expression. J. Cell. Physiol.

[120] Munjal C, Givvimani S, Qipshidze N, Tyagi N, Falcone JC, Tyagi SC (2011). Mesenteric vascular remodeling in hyperhomocysteinemia. Mol. Cell. Biochem.

[121] Schurks M, Rist PM, Kurth T (2010). MTHFR 677C>T and ACE D/I polymorphisms in migraine a systematic review and meta-analysis. Headache.

[122] Leclerc D, Sibani S, Rozen R Molecular Biology of Methylenetetrahydrofolate Reductase (MTHFR) and Overview of Mutations/Polymorphisms. http://www.ncbi.nlm.nih.gov/books/NBK6561/ (accessed June 2013).

[123] Lea R, Colson N, Quinlan S, Macmillan J, Griffiths L (2009). The effects of vitamin supplementation and MTHFR (C677T) genotype on homocysteine-lowering and migraine disability. Pharmacogenet. Genomics.

[124] Spence JDB (2013). vitamin therapy for homocysteine renal function and vitamin B12 determine cardiovascular outcomes. Clin. Chem. Lab. Med.

[125] Wien TN, Pike E, Wisloff T, Staff A, Smeland S, Klemp M (2012). Cancer risk with folic acid supplements a systematic review and meta-analysis. Bmj Open.

[126] Marti-Carvajal AJ, Sola I, Lathyris D, Karakitsiou DE, Simancas-Racines D (2013). Homocysteine-lowering interventions for preventing cardiovascular events. Cochrane Database Syst. Rev.

[127] Huang T, Chen Y, Yang B, Yang J, Wahlqvist ML, Li D (2012). Meta-analysis of B vitamin supplementation on plasma homocysteine cardiovascular and all-cause mortality. Clin. Nutr.

[128] Nachum-Biala Y, Troen AM (2012). B-vitamins for neuroprotection. Narrowing the evidence gap. BioFactors.

[129] Menon S, Lea RA, Roy B, Hanna M, Wee S, Haupt LM, Oliver C, Griffiths LR (2012). Genotypes of the MTHFR C677T and MTRR A66G genes act independently to reduce migraine disability in response to vitamin supplementation. Pharmacogen. Genomics.

[130] Trimmer EE (2013). Methylenetetrahydrofolate Reductase. Biochemical Characterization and Medical Significance. Curr. Pharm. Des.

[131] Lipton RB, Bigal ME, Diamond M, Freitag F, Reed ML, Stewart WF (2007). Migraine prevalence disease burden and the need for preventive therapy. Neurology.

[132] Maggioni F, Alessi C, Maggino T, Zanchin G (1997). Headache during pregnancy. Cephalalgia.

[133] MacGregor EA (2009). Migraine headache in perimenopausal and menopausal women. Cur. Pain Headache Rep.

[134] Sacco S, Ricci S, Degan D, Carolei A (2012). Migraine in women the role of hormones and their impact on vascular diseases. J. Head. Pain.

[135] Shuster LT, Faubion SS, Sood R, Casey PM (2011). Hormonal manipulation strategies in the management of menstrual migraine and other hormonally related headaches. Cur. Neurol. Neurosci. Rep.

[136] Martin VT, Behbehani M (2006). Ovarian hormones and migraine headache. Understanding mechanisms and pathogenesis - Part I. Headache.

[137] Wang XP, Liu JM, Zhao YB (2008). Migraine Sex-influenced trait model. Med. Hypotheses.

[138] Martin VT, Wernke S, Mandell K, Ramadan N, Kao L, Bean J, Liu J, Zoma W, Rebar R (2005). Defining the relationship between ovarian hormones and migraine headache. Headache.

[139] Colson NJ, Lea RA, Quinlan S, MacMillan J, Griffiths LR (2004). The estrogen receptor 1 G594A polymorphism is associated with migraine susceptibility in two independent case/control groups. Neurogenetics.

[140] Lipphardt MF, Deryal M, Ong MF, Schmidt W, Mahlknecht U (2013). ESR1 single nucleotide polymorphisms predict breast cancer susceptibility in the central European Caucasian population. Int. J. Clin. Exp. Med.

[141] Lewis DW, Winner P (2006). The pharmacological treatment options for pediatric migraine: an evidence-based appraisal. NeuroRx.

[142] Gupta S, Mehrotra S, Villalon CM, Perusquia M, Saxena PR, MaassenVanDenBrink A (2007). Potential role of female sex hormones in the pathophysiology of migraine. Pharmacol. Ther.

[143] Kaunisto MA, Kallela M, Hamalainen E, Kilpikari R, Havanka H, Harno H, Nissila M, Sako E, Ilmavirta M, Liukkonen J, Teirmaa H, Tornwall O, Jussila M, Terwilliger J, Farkkila M, Kaprio J, Palotie A, Wessman M (2006). Testing of variants of the MTHFR and ESR1 genes in 1798 Finnish individuals fails to confirm the association with migraine with aura. Cephalalgia.

[144] Oterino A, Pascual J, RuizdeAlegria C, Valle N, Castillo J, Bravo Y, Gonzalez F, Sanchez-Velasco P, Cayon A, Leyva-Cobian F, Alonso-Arranz A, Munoz P (2006). Association of migraine and ESR1 G325C polymorphism. Neuroreport.

[145] Corominas R, Ribases M, Cuenca-Leon E, Cormand B, Macaya A (2009). Lack of association of hormone receptor polymorphisms with migraine. Eur. J. Neurol.

[146] Joshi G, Pradhan S, Mittal B (2010). Role of the oestrogen receptor (ESR1 PvuII and ESR1 325 C->G) and progesterone receptor (PROGINS) polymorphisms in genetic susceptibility to migraine in a North Indian population. Cephalalgia.

[147] Lee H, Sininger L, Jen JC, Cha YH, Baloh RW, Nelson SF (2007). Association of progesterone receptor with migraine-associated vertigo. Neurogenetics.

[148] Colson NJ, Lea RA, Quinlan S, MacMillan J, Griffiths LR (2005). Investigation of hormone receptor genes in migraine. Neurogenetics.

[149] Oterino A, Toriello M, Cayon A, Castillo J, Colas R, Alonson-Arranz A, Ruiz-Alegria C, Quintela E, Monton F, Ruiz-Lavilla N, Gonzalez F, Pascual J (2008). Multilocus analyses reveal involvement of the ESR1 ESR2 and FSHR genes in migraine. Headache.

[150] Schurks M, Rist PM, Kurth T (2010). Sex hormone receptor gene polymorphisms and migraine a systematic review and meta-analysis. Cephalalgia.

[151] Loder E (2005). Menstrual migraine clinical considerations in light of revised diagnostic criteria. Neurol. Sci.

[152] Colson N, Fernandez F, Griffiths L (2010). Genetics of menstrual migraine: the molecular evidence. Cur. Pain Headache Rep.

[153] Hershey A, Horn P, Kabbouche M, O'Brien H, Powers S (2012). Genomic Expression Patterns in Menstrual-Related Migraine in Adolescents. Headache.

[154] Scholte HR (1988). The biochemical basis of mitochondrial diseases. J. Bioenerg. Biomembr.

[155] Reyngoudt H, Achten E, Paemeleire K (2012). Magnetic resonance spectroscopy in migraine. What have we learned so far. Cephalalgia.

[156] Reyngoudt H, Paemeleire K, Descamps B, DeDeene Y, Achten E (2011). 31P-MRS demonstrates a reduction in high-energy phosphates in the occipital lobe of migraine without aura patients. Cephalalgia.

[157] Tanji K, Kunimatsu T, Vu TH, Bonilla E (2001). Neuropathological features of mitochondrial disorders. Semin. Cell Dev. Biol.

[158] Saxena R, deBakker PI, Singer K, Mootha V, Burtt N, Hirschhorn JN, Gaudet D, Isomaa B, Daly MJ, Groop L, Ardlie KG, Altshuler D (2006). Comprehensive association testing of common mitochondrial DNA variation in metabolic disease. Am. J. Hum. Gen.

[159] Montagna P, Sacquegna T, Martinelli P, Cortelli P, Bresolin N, Moggio M, Baldrati A, Riva R, Lugaresi E (1988). Mitochondrial abnormalities in migraine. Preliminary findings. Headache.

[160] Bresolin N, Martinelli P, Barbiroli B, Zaniol P, Ausenda C, Montagna P, Gallanti A, Comi GP, Scarlato G, Lugaresi E (1991). Muscle mitochondrial DNA deletion and 31P-NMR spectroscopy alterations in a migraine patient. J. Neurol. Sci.

[161] Uncini A, Lodi R, DiMuzio A, Silvestri G, Servidei S, Lugaresi A, Iotti S, Zaniol P, Barbiroli B (1995). Abnormal brain and muscle energy metabolism shown by 31P-MRS in familial hemiplegic migraine. J. Neurol. Sci.

[162] Sparaco M, Feleppa M, Lipton RB, Rapoport AM, Bigal ME (2006). Mitochondrial dysfunction and migraine evidence and hypotheses. Cephalalgia.

[163] Sangiorgi S, Mochi M, Riva R, Cortelli P, Monari L, Pierangeli G, Montagna P (1994). Abnormal platelet mitochondrial function in patients affected by migraine with and without aura. Cephalalgia.

[164] Littlewood J, Glover V, Sandler M, Peatfield R, Petty R, CliffordRose F (1984). Low platelet monoamine oxidase activity in headache no correlation with phenolsulphotransferase succinate dehydrogenase platelet preparation method or smoking. J. Neurol. Neurosur. Psychiatry.

[165] D'Andrea G, Leon A (2010). Pathogenesis of migraine from neuro-transmitters to neuromodulators and beyond. Neurol. Sci.

[166] Sandor PS, DiClemente L, Coppola G, Saenger U, Fumal A, Magis D, Seidel L, Agosti RM, Schoenen J (2005). Efficacy of coenzyme Q10 in migraine prophylaxis a randomized controlled trial. Neurology.

[167] Sandor PS, Afra J (2005). Nonpharmacologic treatment of migraine. Cur. Pain Headache Rep.

[168] Pringsheim T, Davenport W, Mackie G, Worthington I, Aube M, Christie SN, Gladstone J, Becker WJ (2012). Canadian Headache Society guideline for migraine prophylaxis. Can. J. Neurol. Sci.

[169] Parikh S, Saneto R, Falk MJ, Anselm I, Cohen BH, Haas R, MedicineSociety TM (2009). A modern approach to the treatment of mitochondrial disease. Cur. Treat. Options in Neurol.

[170] O'Brien HL, Hershey AD (2010). Vitamins and paediatric migraine.Riboflavin as a preventative medication. Cephalalgia.

[171] Reyngoudt H, Paemeleire K, Dierickx A, Descamps B, Vandemaele P, DeDeene Y, Achten E (2011). Does visual cortex lactate increase following photic stimulation in migraine without aura patients.A functional (1)H-MRS study. J. Headache Pain.

[172] Trabka-Janik E, Kulaga A, Urbanik A, Szczudlik A, Grzybek M (2010). Magnetic resonance spectroscopy (HMRS) in migraine. Przeglad lekarski.

[173] Watanabe H, Kuwabara T, Ohkubo M, Tsuji S, Yuasa T (1996). Elevation of cerebral lactate detected by localized 1H-magnetic resonance spectroscopy in migraine during the interictal period. Neurology.

[174] Zaki EA, Freilinger T, Klopstock T, Baldwin EE, Heisner KR, Adams K, Dichgans M, Wagler S, Boles RG (2009). Two common mitochondrial DNA polymorphisms are highly associated with migraine headache and cyclic vomiting syndrome. Cephalalgia.

[175] Majamaa K, Finnila S, Turkka J, Hassinen IE (1998). Mitochondrial DNA haplogroup U as a risk factor for occipital stroke in migraine. Lancet.

[176] Shimomura T, Kitano A, Marukawa H, Mishima K, Isoe K, Adachi Y, Takahashi K (1994). Point mutation in platelet mitochondrial tRNA(Leu(UUR)) in patient with cluster headache. Lancet.

[177] Cortelli P, Zacchini A, Barboni P, Malpassi P, Carelli V, Montagna P (1995). Lack of association between mitochondrial tRNA(Leu(UUR)) point mutation and cluster headache. Lancet.

[178] Klopstock T, May A, Seibel P, Papagiannuli E, Diener HC, Reichmann H (1996). Mitochondrial DNA in migraine with aura. Neurology.

[179] Seibel P, Grunewald T, Gundolla A, Diener HC, Reichmann H (1996). Investigation on the mitochondrial transfer RNA(Leu(UUR) in blood cells from patients with cluster headache. J. Neurol.

[180] Haan J, Terwindt GM, Maassen JA, Hart LM, Frants RR, Ferrari MD (1999). Search for mitochondrial DNA mutations in migraine subgroups. Cephalalgia.

[181] Lewis SN, Nsoesie E, Weeks C, Qiao D, Zhang L (2011). Prediction of disease and phenotype associations from genome-wide association studies. PLoS One.

[182] Lindquist KJ, Jorgenson E, Hoffmann TJ, Witte JS (2013). The impact of improved microarray coverage and larger sample sizes on future genome-wide association studies. Genet. Epidemiol.

[183] Anttila V, Stefansson H, Kallela M, Todt U, Terwindt GM, Calafato MS, Nyholt DR, Dimas AS, Freilinger T, Muller-Myhsok B, Artto V, Inouye M, Alakurtti K, Kaunisto MA, Hamalainen E, deVries B, Stam AH, Weller CM, Heinze A, Heinze-Kuhn K, Goebel I, Borck G, Gobel H, Steinberg S, Wolf C, Bjornsson A, Gudmundsson G, Kirchmann M, Hauge A, Werge T, Schoenen J, Eriksson JG, Hagen K, Stovner L, Wichmann HE, Meitinger T, Alexander M, Moebus S, Schreiber S, Aulchenko YS, Breteler MM, Uitterlinden AG, Hofman A, vanDuijn CM, Tikka-Kleemola P, Vepsalainen S, Lucae S, Tozzi F, Muglia P, Barrett J, Kaprio J, Farkkila M, Peltonen L, Stefansson K, Zwart JA, Ferrari MD, Olesen J, Daly M, Wessman M, vandenMaagdenberg AM, Dichgans M, Kubisch C, Dermitzakis ET, Frants RR, Palotie A (2010). Genome-wide association study of migraine implicates a common susceptibility variant on 8q22. . Nat. Gen.

[184] Ligthart L, deVries B, Smith AV, Ikram MA, Amin N, Hottenga JJ, Koelewijn SC, Kattenberg VM, deMoor MH, Janssens AC, Aulchenko YS, Oostra BA, deGeus EJ, Smit JH, Zitman FG, Uitterlinden AG, Hofman A, Willemsen G, Nyholt DR, Montgomery G W, Terwindt GM, Gudnason V, Penninx BW, Breteler M, Ferrari MD, Launer LJ, vanDuijn CM, vandenMaagdenberg AM, Boomsma DI (2011). Meta-analysis of genome-wide association for migraine in six population-based European cohorts. Europ. J. Hum. Genet.

[185] Chasman DI, Schurks M, Anttila V, deVries B, Schminke U, Launer LJ, Terwindt GM, vandenMaagdenberg AM, Fendrich K, Volzke H, Ernst F, Griffiths LR, Buring JE, Kallela M, Freilinger T, Kubisch C, Ridker PM, Palotie A, Ferrari MD, Hoffmann W, Zee RY, Kurth T (2011). Genome-wide association study reveals three susceptibility loci for common migraine in the general population. Nat. Gen.

[186] Freilinger T, Anttila V, deVries B, Malik R, Kallela M, Terwindt GM, Pozo-Rosich P, Winsvold B, Nyholt DR, vanOosterhout WP, Artto V, Todt U, Hamalainen E, Fernandez-Morales J, Louter MA, Kaunisto MA, Schoenen J, Raitakari O, Lehtimaki T, Vila-Pueyo M, Gobel H, Wichmann E, Sintas C, Uitterlinden AG, Hofman A, Rivadeneira F, Heinze A, Tronvik E, vanDuijn CM, Kaprio J, Cormand B, Wessman M, Frants RR, Meitinger T, Muller-Myhsok B, Zwart JA, Farkkila M, Macaya A, Ferrari MD, Kubisch C, Palotie A, Dichgans M, vandenMaagdenberg AM (2012). Genome-wide association analysis identifies susceptibility loci for migraine without aura. Nat. Gen.

